# Prevalence of gout in the Sincan district of Ankara, Türkiye: a primary care-based cross-sectional study

**DOI:** 10.55730/1300-0144.6134

**Published:** 2025-12-21

**Authors:** Rıza Can KARDAŞ, Yasemin ÜNSAL, Derya YILDIRIM, Hakan TÜZÜN, Mustafa Necmi İLHAN, Hamit KÜÇÜK, Abdurrahman TUFAN, M. Akif ÖZTÜRK

**Affiliations:** 1 Division of Rheumatology, Department of Internal Medicine, Faculty of Medicine, Gazi University, Ankara, Turkiye; 2 Department of Internal Medicine, Faculty of Medicine, Gazi University, Ankara, Turkiye; 3 Department of Public Health, Faculty of Medicine, Gazi University, Ankara, Turkiye

**Keywords:** Gout, prevalence, epidemiology, Türkiye, Ankara, community-based study

## Abstract

**Background/aim:**

Gout, the most prevalent form of inflammatory arthritis, has shown a global increase in prevalence; however, data from Türkiye remain scarce, inconsistent, and largely confined to western regions. This study aimed to estimate the prevalence of gout among adults in the Sincan district of Ankara, Türkiye, and to provide updated data from a previously understudied region.

**Materials and methods:**

This cross-sectional, community-based study employed a multistage design. Adults aged ≥18 years who presented to selected family health centers in Sincan between February and June 2023 were screened using a structured questionnaire. Individuals with suspected gout underwent comprehensive clinical evaluation, including medical history, physical examination, and relevant laboratory testing, conducted by rheumatologists. Gout was classified according to the 2015 ACR/EULAR criteria, with a score of ≥8 required for classification.

**Results:**

Of the 517 individuals approached, 515 provided consent, and data from 513 participants were included in the final analysis. Screening identified 26 individuals with suspected gout, of whom 24 completed evaluation, resulting in the confirmation of seven gout cases (five men and two women). The overall estimated prevalence of gout was 1.36% (95% CI: 0.55%–2.81%). Prevalence was higher in men (2.18%; 95% CI: 0.71%–5.01%) than in women (0.70%; 95% CI: 0.09%–2.52%) although this difference was not statistically significant due to the small sample size, and prevalence increased with age.

**Conclusion:**

These findings provide updated epidemiological data, indicating a higher prevalence than previously reported in western Türkiye, while remaining within the lower range of global estimates. These data underscore the need to enhance gout awareness and to develop effective management strategies within the Turkish population.

## 1. Introduction

Gout represents the most prevalent form of inflammatory arthritis globally. It is characterized by the deposition of monosodium urate (MSU) crystals within the joints and soft tissues, secondary to persistent hyperuricemia [1]. Clinically, gout typically presents with recurrent acute attacks of intense joint pain and inflammation, most frequently affecting the first metatarsophalangeal joint, and may progress to chronic polyarticular arthritis with tophus formation. Historically referred to as the “disease of kings” [2], gout has shown a marked global increase in prevalence over recent decades, largely driven by rising rates of obesity, metabolic syndrome, and dietary transitions [3–5]. Global prevalence estimates vary widely, typically ranging from below 1% to as high as 10% [3–5], and gout imposes a considerable burden on healthcare systems due to its comorbidities and increased healthcare utilization [5,6].

Accurate epidemiological data are essential for quantifying disease burden, guiding resource allocation, informing clinical guideline development, and shaping public health initiatives [4,7]. Misconceptions regarding disease prevalence may result in diagnostic errors and suboptimal patient management [8–10]. For instance, underestimating the prevalence of gout may lead clinicians to overlook the diagnosis, particularly in patients with atypical presentations, thereby delaying treatment and increasing the risk of chronic complications such as joint damage. Conversely, gout may be overdiagnosed in primary care when nonspecific joint pain or isolated hyperuricemia is misinterpreted as gout, thereby inflating prevalence estimates. The evolving nature of gout epidemiology necessitates periodic reassessment of its prevalence and associated risk factors across diverse populations [5,11].

In Türkiye, existing data on gout prevalence are limited and show inconsistent findings. Previous research includes two population-based studies conducted in western Türkiye, which reported markedly different prevalence rates. A 2012 study conducted in Havsa (Edirne province) reported a prevalence of 0.018% [12], whereas a 2014 study from the Balçova and Narlıdere districts of İzmir found an age- and sex-adjusted prevalence of 0.31% [13], approximately 15 times higher than that reported in the Havsa study. This substantial discrepancy may result from methodological differences, regional variations in lifestyle or genetic background, or temporal shifts in disease prevalence. Supporting the likelihood of regional variation, the prevalence of uric acid nephrolithiasis in Türkiye has been reported to be higher in inland regions than in coastal areas [14], suggesting possible geographic disparities in hyperuricemia patterns. These conflicting findings and the geographical limitation of previous studies underscore the need for updated, nationwide epidemiological data on gout in Türkiye.

Ankara, the capital city of Türkiye, attracts substantial internal migration from diverse regions nationwide [15,16]. The Sincan district, a large and densely populated metropolitan area within Ankara (approximately 580,000 residents), exhibits considerable socioeconomic diversity and may reflect broader demographic trends among urban Turkish populations [17]. Its relatively young population (median age of 30 years)^1^ and the coexistence of traditional and modern lifestyles provide a unique opportunity to investigate gout within this setting. Estimating the prevalence of gout in this cosmopolitan district may yield valuable insights into the current disease burden in a previously unstudied major region and may inform healthcare planning within Türkiye’s universal healthcare system.

Accordingly, the primary objective of this community-based, cross-sectional study was to estimate the prevalence of gout among adults residing in the Sincan district of Ankara. A multistage approach was adopted, comprising an initial screening phase at primary care centers, followed by comprehensive clinical evaluation and application of the 2015 ACR/EULAR classification criteria for case confirmation. Secondary objectives were to describe the clinical characteristics of individuals diagnosed with gout and to assess their level of diagnostic awareness.

## 2. Materials and methods

### 2.1. Study design and setting

**Figure 1:**
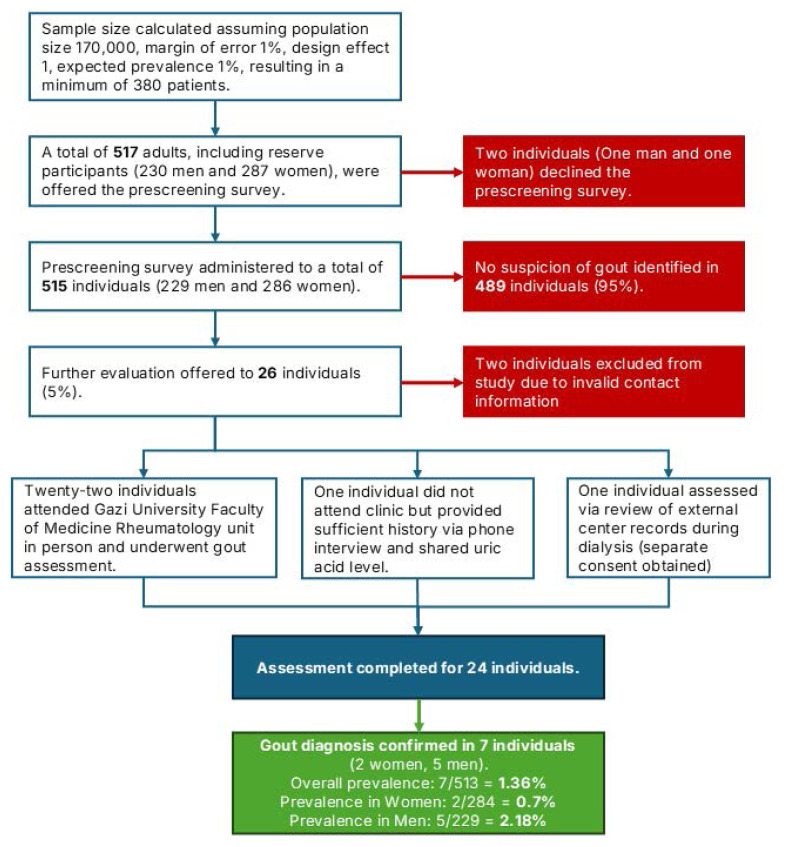
Flow diagram illustrating participant recruitment, screening, eligibility assessment, and inclusion in the gout prevalence study.

This cross-sectional, community-based study adopted a multistage design to estimate the prevalence of gout among adults residing in the Sincan district of Ankara, Türkiye. The study was conducted between February and June 2023. Data collection was carried out in two stages: an initial screening phase conducted at selected family health centers (Aile Sağlığı Merkezleri, ASMs) within the district, followed by a detailed clinical evaluation of individuals with suspected gout at the Department of Rheumatology, Gazi University Faculty of Medicine (Figure 1).

### 2.2. Study population and sampling strategy

The target population consisted of adults residing in the Sincan district of Ankara, Türkiye. Stage 1 participants were recruited using a convenience sampling approach from individuals who presented for any reason to four participating ASMs. The required sample size was calculated assuming a gout prevalence of 1%, a 1% margin of error, and a design effect of 1.0, resulting in a minimum sample of 384 participants. To account for an anticipated 30% nonresponse rate, the target sample size was increased to 494 participants.

Inclusion criteria for stage 1 included being aged ≥18 years and providing written informed consent. Individuals eligible for stage 2 were excluded if contact could not be established after three attempts (by telephone or email), if the provided contact information was invalid, or if informed consent was withdrawn.

### 2.3. Data collection: stage 1 – screening phase

During stage 1, trained medical interns (n = 15), under the supervision of faculty members from the Departments of Public Health and Rheumatology, administered a structured screening questionnaire (Supplementary File) to eligible participants at the ASMs. This questionnaire was specifically designed to identify individuals with suspected gout. The key items assessed in the questionnaire included the following:

- History of physician-diagnosed gout.
- Previous prescriptions for gout-specific medications.
- Episodes of sudden-onset, atraumatic joint pain, particularly involving the foot (e.g., first metatarsophalangeal joint and midfoot) or the ankle.
- Characteristics of inflammatory episodes, including the specific joint involved, presence of erythema, severe tenderness (limiting touch or weight-bearing), time to peak pain intensity (<24 h), and resolution within approximately 14 days.

Participants who reported a previous physician diagnosis of gout or symptoms suggestive of the disease were classified as suspected cases and invited to stage 2 for further evaluation.

### 2.4. Data collection: stage 2 – clinical evaluation

Individuals classified as suspected gout cases were contacted by telephone and invited to undergo clinical evaluation at the Gazi University Rheumatology Clinic. To facilitate participation, flexible appointment options, including evenings and weekends, were made available. All clinical assessments were performed by, or under the direct supervision of, experienced rheumatologists.

The clinical assessment protocol consisted of the following components:

- A comprehensive medical history focusing on joint-related symptoms, featured of suspected gout flares, comorbidities, current medications, and relevant family history.
- A focused physical examination emphasizing musculoskeletal findings, including systematic assessment for tophi (e.g., auricular helix, olecranon bursae, digits, and tendons).
- Laboratory investigations were performed as clinically indicated, serum uric acid (sUA), complete blood count (CBC), serum creatinine (with estimated glomerular filtration rate [eGFR]), liver function tests (aspartate aminotransferase [AST], alanine aminotransferase [ALT], gamma-glutamyl transferase [GGT]), lipid profile, fasting plasma glucose, glycated hemoglobin (HbA1c), erythrocyte sedimentation rate (ESR), C-reactive protein (CRP), ferritin, and transferrin saturation.
- Imaging and synovial fluid assessment: Plain radiographs of symptomatic joints were obtained when clinically indicated, and joint ultrasonography was performed as needed. Arthrocentesis for synovial fluid analysis to identify monosodium urate (MSU) crystals was not performed, as the diagnosis of crystal arthropathy was considered clinically certain based on other available clinical and imaging data, thereby obviating the need for microscopic confirmation in this cohort.

For participants unable to attend the clinic in person, a detailed medical history was obtained through a structured telephone interview. With explicit participant consent, relevant existing laboratory and imaging data were retrieved from the national electronic health record system (e-Nabız, Ministry of Health, Türkiye).

### 2.5. Case definition and diagnostic criteria for gout

Gout was classified according to the 2015 American College of Rheumatology/European League Against Rheumatism (ACR/EULAR) classification criteria [18]. Considering the epidemiological setting and the impracticality of performing routine synovial fluid analysis for monosodium urate (MSU) crystal confirmation—the diagnostic gold standard—these validated criteria were applied. Individuals who achieved a total score of ≥8 points based on these criteria were classified as having gout.

### 2.6. Statistical analysis

Descriptive statistics were applied to summarize the demographic and clinical characteristics of the participants. Continuous variables were assessed for normality. Normally distributed data are presented as mean ± standard deviation (SD), whereas nonnormally distributed data are reported as median (range). Categorical variables are presented as frequencies (n) and percentages (%).

Prevalence estimates of gout (overall, age-stratified, and sex-specific) were calculated by dividing the number of participants classified as having gout, according to the ACR/EULAR criteria, by the total number of participants successfully screened in stage 1 (n = 513; representing 515 initially screened individuals minus two lost to follow-up). Exact 95% confidence intervals (CIs) for the prevalence estimates were calculated using the Clopper–Pearson binomial method. All statistical analyses were performed using IBM SPSS Statistics for Windows, version 26.0 (IBM Corp., Armonk, NY, USA).

## 3. Results

### 3.1. Participant recruitment and flow

During the screening phase conducted at four ASMs in Sincan, Ankara, 517 individuals were approached for participation. Of these, 515 individuals (99.6%) provided informed consent and completed the screening questionnaire (Figure 1). The screened cohort comprised 515 participants, of whom 55.5% were women. The mean age of participants was 46.3 ± 15.2 years. Detailed demographic characteristics of the study population are presented in Table 1.

Screening identified 26 individuals (5.0%) as suspected gout cases. Participants were excluded if their contact information was invalid (n = 2). Consequently, 24 individuals (12 men and 12 women) underwent comprehensive clinical evaluation, corresponding to a follow-up rate of 92.3%. Clinical assessments were performed in person for 22 participants, while for the remaining two, evaluations were conducted via telephone interview combined with review of their electronic health records.

### 3.2. Prevalence of gout

Among the 24 individuals who underwent clinical evaluation in stage 2, seven participants (five men and two women) fulfilled the 2015 ACR/EULAR classification criteria for gout. Based on these seven confirmed cases identified within the evaluable screened cohort (n = 513), the overall prevalence of gout was estimated at 1.36% (95% CI: 0.55%–2.81%).

**Figure 2:**
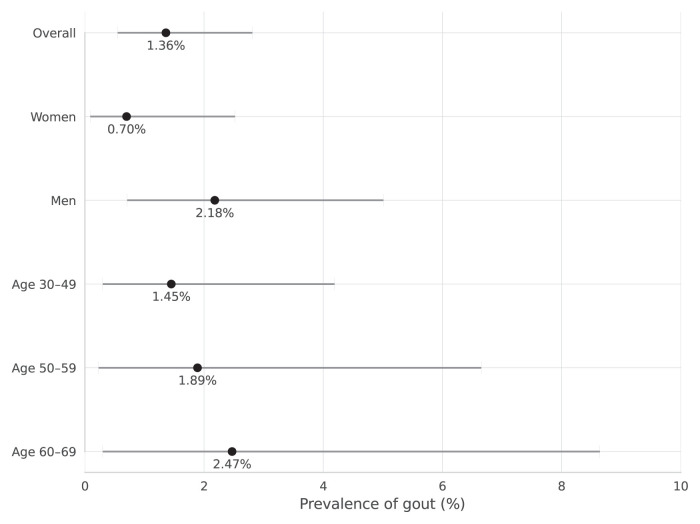
Dot plot showing gout prevalence with 95% confidence intervals by overall, sex, and age group. The highest prevalence was observed among men (2.18%) and participants aged 60–69 years (2.47%). The overall prevalence in the study population was 1.36%. Confidence intervals were calculated using the Clopper–Pearson method. No cases were identified among participants under 30 or over 70 years of age; therefore, these subgroups were excluded from the figure.

The prevalence of gout was higher among men (2.18%; 95% CI: 0.71%–5.01%) than among women (0.70%; 95% CI: 0.09%–2.52%). Age stratification revealed a progressive increase in prevalence with advancing age, ranging from 0% among participants aged 20–29 years to 2.5% among those aged ≥70 years (Table 2; Figure 2).

### 3.3. Demographic and clinical characteristics of participants with confirmed gout

All participants with confirmed gout reported typical acute inflammatory flares characterized by marked tenderness, erythema, and functional limitation, with rapid onset in most cases. The first metatarsophalangeal joint was the most frequently affected site. Metabolic risk factors were prevalent, with most individuals being overweight and several reporting a high intake of dietary meat. Diabetes mellitus and nephrolithiasis were the most frequent observed comorbidities.

In terms of disease management, most patients reported a previous diagnosis or history of treatment for gout. However, at the time of assessment, only a minority were receiving urate-lowering therapy, while colchicine use was comparatively more common. Detailed clinical and laboratory characteristics of patients with confirmed gout are presented in Table 3.

## 4. Discussion

### 4.1. Main findings and study context

Epidemiological data on gout in Türkiye have historically been confined to specific western regions, leaving major knowledge gaps concerning the disease burden in central Anatolia and within the primary care setting. Addressing this gap, the present study provides the first prevalence estimate for gout in the Sincan district of Ankara. We observed an overall gout prevalence of 1.36% (95% CI: 0.55%–2.81%). Consistent with global gout epidemiology, the prevalence was higher among men (2.18%) than among women (0.70%) and increased with age [19,20].

Our prevalence estimate is markedly higher than those reported in previous population-based studies from western Türkiye [12,13]. Although this difference may partly reflect a genuine increase in gout prevalence driven by population aging and urbanization [3,19,20], methodological differences are likely a major contributing factor. In contrast to previous Turkish studies that relied on general rheumatology surveys or self-reported data, our study employed a multistage design with rigorous clinical validation by rheumatologists based on the 2015 ACR/EULAR criteria [18]. This targeted methodological approach likely improved case detection and diagnostic accuracy, as nearly all confirmed cases in our study had a previous history of gout diagnosis or treatment. Additionally, regional factors may contribute, as inland regions of Türkiye have been reported to exhibit higher rates of uric acid–related conditions than coastal areas [14,21].

### 4.2. Global context, dietary factors, and public health implications

Internationally, the observed prevalence of 1.36% positions this urban Turkish population at an intermediate level–it is higher than rates typically reported in developing nations, yet lower than those observed in high-income Western countries [4,6,20,22]. This likely reflects Türkiye’s ongoing socioeconomic and nutritional transition [4].

Dietary changes in urban Türkiye are likely a key driver of this epidemiological pattern. Although traditional dietary patterns may have historically provided some protection [23,24], ongoing urbanization has contributed to increased consumption of processed foods, saturated fats, and refined carbohydrates [3,25]. These changes, characteristic of the global nutrition transition, are closely associated with the increasing prevalence of metabolic syndrome and gout. Consequently, incorporating gout prevention into broader public health strategies targeting noncommunicable diseases and obesity has become increasingly important for Türkiye [4].

### 4.3. Clinical profile and comorbidities among gout cases

The clinical profile of confirmed gout cases in our study was consistent with established global and national patterns [4,6,26–28]. The majority of patients were middle-aged men, and involvement of the first metatarsophalangeal joint was common. Comorbidities were common, particularly obesity, diabetes mellitus, and nephrolithiasis, underscoring the strong association between gout and metabolic syndrome [4]. This clustering of risk factors is particularly relevant in the Turkish context, where the prevalence of metabolic syndrome is notably high [24]. Given these findings, a diagnosis of gout in the primary care setting should be considered a sentinel event, prompting routine and comprehensive cardiometabolic risk assessment to identify and manage underlying comorbidities.

Additional observations warrant comment on two notable points. First, the low rate of reported family history (14.3%) suggests possible underdiagnosis or limited disease awareness among previous generations. Second, the absence of clinically apparent tophi, despite a median disease duration of 5 years, is consistent with findings from other Turkish cohorts [26] and may reflect intermittent treatment or distinct disease phenotypes.

### 4.4. Therapeutic gaps and challenges in gout management

Our findings underscore significant gaps in the long-term management of gout. Although most patients had a previous diagnosis, fewer than half were receiving urate-lowering therapy at the time of assessment, whereas colchicine use was comparatively more common. This pattern indicates a therapeutic emphasis on managing acute flares rather than maintaining long-term urate control—a trend frequently reported both in Türkiye [26,28] and internationally [4]. Addressing this “therapeutic inertia” necessitates enhanced provider education on treat-to-target strategies, alongside improved patient counseling regarding the chronic nature of the disease [6,29].

### 4.5. Study limitations and strengths

Our findings should be interpreted in the context of certain limitations. Recruitment through family health centers may have introduced selection bias, as individuals seeking healthcare could have a higher burden of comorbidities than the general population. Although these centers also provide preventive services, this recruitment strategy could potentially inflate prevalence estimates relative to random household sampling. Furthermore, as the study was limited to a single urban district, the findings may not be generalizable to rural settings or other geographic regions. The relatively small number of confirmed cases limits the statistical precision of subgroup analyses. Detailed data on lifestyle variables—such as alcohol type and quantity, quantitative purine intake, and physical activity—were not collected; future studies should incorporate these assessments to better characterize regional risk profiles. Finally, the diagnosis was based on clinical classification criteria rather than on synovial fluid analysis. Although standard in epidemiological research, this approach lacks the absolute diagnostic certainty provided by crystal identification; however, the use of validated criteria by trained rheumatologists likely ensured high diagnostic specificity.

Despite these limitations, the study possesses several notable strengths. It is the first study to estimate gout prevalence in central Türkiye using a multistage design that combines community-based screening with rheumatologist-led clinical confirmation. The high follow-up rate among screen-positive individuals (92.3%) enhances the study’s internal validity. This methodological approach represents a substantial improvement in diagnostic accuracy over previous survey-based studies and provides essential baseline data for this population.

## 5. Conclusion

This study provides the first estimate of gout prevalence in central Türkiye, identifying a prevalence rate of 1.36% among adults attending urban primary care centers in Ankara. This prevalence is markedly higher than previously reported national estimates and underscores the increasing public health importance of gout in the region. The clinical profile of affected individuals emphasizes the need for integrated care models that address both articular manifestations and associated cardiometabolic comorbidities. Enhancing provider adherence to long-term urate-lowering therapy and integrating routine cardiovascular risk assessment into gout management represent essential next steps.

## Supplementary File: Gout Screening Questionnaire

(Administered at Family Health Centers - ASMs)

1. **Daha önce bir doktor tarafından gut hastası olduğunuz söylendi mi?**
  □ Evet
  □ Hayır
2. **Daha önce gut hastalığına yönelik ilaç tedavisi reçete edildi mi?**
  □ Evet
  □ Hayır
3. **Ayak başparmağı, tarak kemikleri ya da ayak bileğinizde travma olmadan ani başlangıçlı ağrı yaşadınız mı?**
  □ Evet
  □ Hayır (Eğer Hayır ise, sona atlayınız)
4. **Eğer Soru 3’e evet yanıtı verdiyseniz, ayağınızın hangi kısmında ağrı/kızarıklık/şişlik oldu?** (Uygulanan tüm seçenekleri işaretleyiniz)
  □ Ayak başparmağı
  □ Ayak tabanı-kenarı
  □ Ayak bileği
  □ Diğer (Lütfen belirtiniz): ______________
5. **Soru 3’te belirtilen ağrı atağı/atakları ile ilgili olarak, aşağıdaki özelliklerden herhangi birini yaşadınız mı?** (Uygulanan tüm seçenekleri işaretleyiniz)
  □ Etkilenen eklem üzerinde kızarıklık
  □ Etkilenen eklemi kullanamama/yürüyememe/dokunmakla ciddi hassasiyet durumlarından biri
6. **Soru 3’te belirtilen ağrı atağı/atakları ile ilgili olarak, ağrı en şiddetli haline 24 saat içinde mi ulaştı?**
  □ Evet
  □ Hayır
  □ Emin değilim
7. **Soru 3’te belirtilen ağrı atağı/atakları ile ilgili olarak, ağrı 2 hafta içinde kendiliğinden (veya basit ağrı kesicilerle) tamamen geçti mi?**
  □ Evet
  □ Hayır
  □ Emin değilim

## Figures and Tables

**Table 1:** Demographic characteristics of the screened population with successful follow-up (n = 513).

Characteristic	Overall (n=513)	Men (n=229)	Women (n=284)
**Age**, years, mean ± SD	46.3 ± 15.2	48.9 ± 16.4	44.3 ± 13.8
**Age group**, n (%)			
20–29	85 (16.6%)	38 (16.6%)	47 (16.5%)
30–39	97 (18.9%)	36 (15.7%)	61 (21.5%)
40–49	110 (21.4%)	34 (14.8%)	76 (26.8%)
50–59	106 (20.7%)	49 (21.4%)	57 (20.1%)
60–69	81 (15.8%)	52 (22.7%)	29 (10.2%)
70–79	28 (5.5%)	15 (6.6%)	13 (4.6%)
≥80	7 (1.4%)	6 (2.6%)	1 (0.4%)

**Table 2:** Overall and stratified prevalence of gout by sex and age group (n = 513).

Subgroup	Confirmed cases/total in subgroup	Prevalence, % (95% CI)
**Overall**	7/513	1.36 (0.55–2.81)
**Sex**		
Men	5/229	2.18 (0.71–5.01)
Women	2/284	0.70 (0.09–2.52)
**Age group** (years)		
18–30	0/85	–
30–49	3/207	1.45 (0.30–4.19)
50–59	2/106	1.89 (0.23–6.65)
60–69	2/81	2.47 (0.30–8.64)
≥70	0/35	–

**Table 3:** Demographic, clinical, and laboratory characteristics of patients with confirmed gout (n = 7).

Characteristic	Value
**Demographics**	
Age, years, median (min–max)	55 (36–64)
Sex, n (%)	
Male	5 (71.4%)
Female	2 (28.6%)
Body mass index, kg/m^2^, mean ± SD	29.7 ± 3.85
**Clinical features**	
Disease duration since first attack, years, median (IQR)	5 (2–8)
Subcutaneous tophi present, n (%)	0 (0.0%)
First MTP joint involvement (ever), n (%)	5 (71.4%)
Oligoarticular attacks (ever), n (%)	5 (71.4%)
**Comorbidities**, n (%)	
Hypertension	1 (14.3%)
Diabetes mellitus	2 (28.6%)
Nephrolithiasis	2 (28.6%)
**Lifestyle factors**, n (%)	
Current smoking	1 (14.3%)
Alcohol consumption	2 (28.6%)
Excessive meat consumption	3 (42.9%)
**Family history**, n (%)	
Gout	1 (14.3%)
Coronary artery disease	5 (71.4%)
Diabetes mellitus	4 (57.1%)
Hypertension	2 (28.6%)
**Laboratory findings**, median (min–max)	
Serum uric acid, mg/dL*	7.2 (3.9–10.9)
Serum creatinine, mg/dL	0.9 (0.8–1.1)
ESR, mm/h	18 (7–42)
CRP, mg/L	6.37 (2.82–9.9)
HbA1c, %	5.9 (5.6–8.8)
Triglycerides, mg/dL	208 (98–1395)
LDL cholesterol, mg/dL	120 (103–174)
HDL cholesterol, mg/dL	37 (25–58)
**Treatment status**, n (%)	
Prior gout diagnosis or prescription	6 (85.7%)
Current allopurinol use	3 (42.9%)
Current colchicine use	5 (71.4%)
Recent NSAID use	2 (28.6%)
Current diuretic use	0 (0.0%)

CRP: C-reactive protein; ESR: erythrocyte sedimentation rate; HbA1c: glycated hemoglobin; HDL: high-density lipoprotein; LDL: low-density lipoprotein; Min–Max: minimum–maximum range; MTP: metatarsophalangeal; NSAID: nonsteroidal antiinflammatory drug; SD: standard deviation.

* Three patients were receiving allopurinol at the time of measurement.

## Footnotes

1 Türkiye İstatistik Kurumu (2023). Adrese Dayalı Nüfus Kayıt Sistemi Sonuçları, 2023 [online]. Website https://data.tuik.gov.tr/Bulten/Index?p=Adrese-Dayali-Nufus-Kayita-Sistemi-Sonuclari-2023-49684 [accessed 12 June 2024] (in Turkish).
